# SUPER AGE: THE RISE OF BIOSOCIAL SUPER AGE ESTIMATES AS ALTERNATIVES TO CHRONOLOGICAL AGE

**DOI:** 10.1093/geroni/igae098.3423

**Published:** 2024-12-31

**Authors:** Adam Perzynski, Douglas Gunzler, Jarrod Dalton, Kristen Berg

**Affiliations:** MetroHealth and Case Western Reserve University, Cleveland, Ohio, United States; CWRU, Cleveland, Ohio, United States; Cleveland Clinic, Cleveland, Ohio, United States; CWRU and MetroHealth, Cleveland, Ohio, United States

## Abstract

Recent discoveries have established conceptual and empirical linkages between the social characteristics of communities, biological and physiological variation and disease onset and outcomes. Cellular and molecular markers appear to be at least partially indicative of an accelerated aging process. We are approaching the widespread availability of “Super Ages”, highly accurate biosocial estimates of the aging of a human person. Super Ages have more predictive validity for disease and disability than chronological age. As compared with chronological age, Super Age estimates can be described as having an accelerated (hyper-aging) or decelerated (hypo-aging) character. We ask: What types of social changes– beneficial or detrimental– might be postulated based upon a shift toward incorporating biosocial measures of aging in age-graded policy and care delivery? We present case-based scenarios illustrative of the coming biosocial dilemma. Consider two matched neighborhoods each containing 400 adults with a chronological age between 40 and 50 years who have aged in place (Neighborhood A and Neighborhood B). All persons in Neighborhood A experience uniform social and environmental hazards while hazards are minimal in Neighborhood B. Molecular analyses find Neighborhood A adults experience hyper-aging of chronological age plus 4 to 7 years. Neighborhood B are as much as 3 to 5 years “younger” creating between community biosocial age gap of 9.5 years. Both neighborhoods experience equal access to health services. Guideline-based screening (i.e. breast, lung and colon cancer screening) is age-graded but Neighborhood A is more vulnerable. Super Age estimates are fraught with new policy and care delivery challenges.

